# Conditional Cooperativity in DNA Minor-Groove Recognition by Oligopeptides

**DOI:** 10.3390/molecules26175188

**Published:** 2021-08-26

**Authors:** Jurij Lah, San Hadži

**Affiliations:** Faculty of Chemistry and Chemical Technology, University of Ljubljana, Večna Pot 113, 1000 Ljubljana, Slovenia

**Keywords:** DNA recognition, conditional cooperativity, transcription regulation, protein–DNA interactions, ligand–DNA interactions, distamycin, netropsin, thermodynamics of binding, phase diagrams, DNA folding

## Abstract

The recognition of specific DNA sequences in processes such as transcription is associated with a cooperative binding of proteins. Some transcription regulation mechanisms involve additional proteins that can influence the binding cooperativity by acting as corepressors or coactivators. In a conditional cooperativity mechanism, the same protein can induce binding cooperativity at one concentration and inhibit it at another. Here, we use calorimetric (ITC) and spectroscopic (UV, CD) experiments to show that such conditional cooperativity can also be achieved by the small DNA-directed oligopeptides distamycin and netropsin. Using a global thermodynamic analysis of the observed binding and (un)folding processes, we calculate the phase diagrams for this system, which show that distamycin binding cooperativity is more pronounced at lower temperatures and can be first induced and then reduced by increasing the netropsin or/and Na+ ion concentration. A molecular interpretation of this phenomenon is suggested.

## 1. Introduction

The specific recognition of DNA by proteins is critical for the regulation of physiological processes such as DNA transcription and replication. Their precise regulation requires the cooperative association of DNA-binding proteins with other proteins that can act as transcription or replication co- or de-repressors.

About a decade ago, an interesting mechanism for the regulation of transcription was discovered in which a particular protein can act as a co- and de-repressor [1,2,3,4]. This phenomenon, termed conditional cooperativity, depends on the concentration of the protein relative to another specific DNA-binding protein and is achieved by linkage of the two proteins via steric, allosteric, and/or avidity effects [5].

Protein binding to DNA can be influenced by small molecules (ligands) that bind to specific DNA sequences. For example, some ligands are used as drugs that inhibit transcription or replication. In addition, they can be used as fluorescent probes to monitor specific parts of DNA and for basic purposes to study the possible molecular mechanisms of DNA recognition [6,7,8,9].

**Scheme 1:**
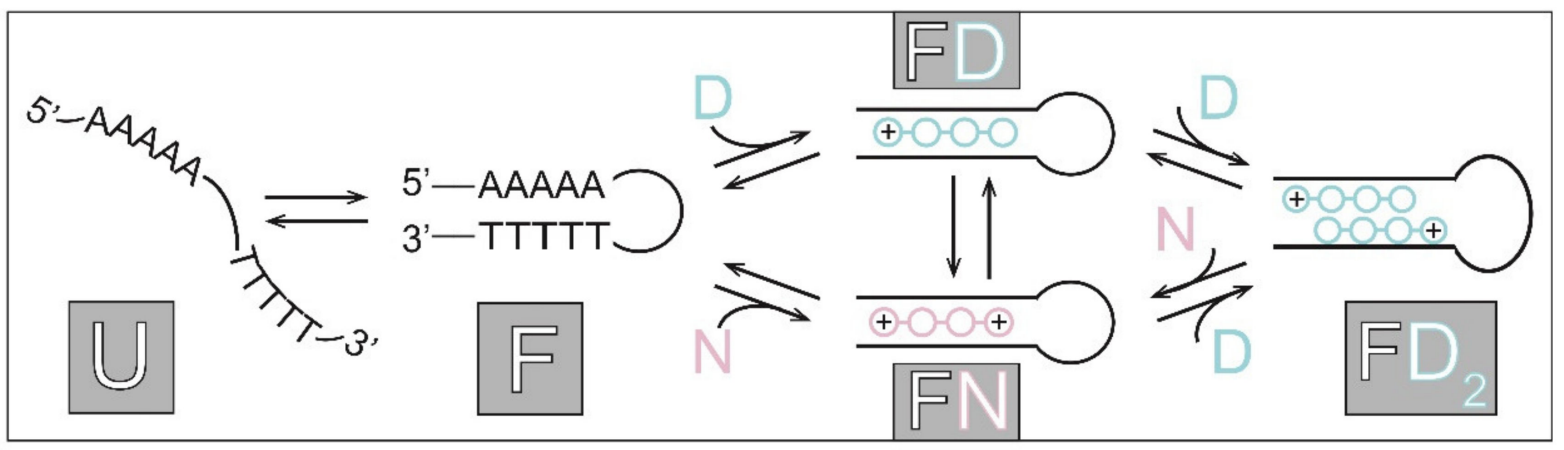
Mechanism of 5′-GAAAAACCCCCTTTTTC-3′ folding and binding of distamycin (D) and netropsin (N) to the folded oligonucleotide (F).

A well-known class of DNA-directed ligands are oligopeptides that bind to the minor groove of DNA [10,11,12]. Numerous studies focus on two natural antibiotics, the singly charged distamycin A (D) and the doubly charged netropsin (N), which bind predominantly to the AT-rich sequences of four to five base pairs (Scheme 1). Both ligands exhibit strong 1:1 binding. In addition, distamycin can form 2:1 complexes in which the bound DST molecules are stacked in a head-to-tail orientation with the charged groups located at opposite ends of the complex. It is known that the binding cooperativity of the two distamycin molecules strongly depends on the sequence [13,14,15,16,17,18,19,20,21]. However, the aim of this study is to investigate how the presence of another ligand (netropsin) affects the cooperative distamycin binding. In other words, how linkage by multiple binding equilibria allows conditional cooperativity, where netropsin binding would induce the formation of distamycin 2:1 complexes, and how these linked equilibria are affected by temperature and salt concentration.

To address these questions, we characterized the binding of netropsin and distamycin to a hairpin-forming oligonucleotide containing the AAAAA binding site (Scheme 1). Binding was monitored directly by isothermal titration calorimetry and CD spectroscopy and indirectly by ligand-induced stabilization of the model duplex by UV melting experiments. Model analysis of the obtained data led to thermodynamic parameters of DNA folding and ligand binding. These parameters were used to predict the DNA stability phase space, which is presented in the form of phase diagrams showing how distamycin binding cooperativity is conditioned by solution conditions.

## 2. Results and Discussion

### 2.1. Thermodynamics of Binding

**Figure 1:**
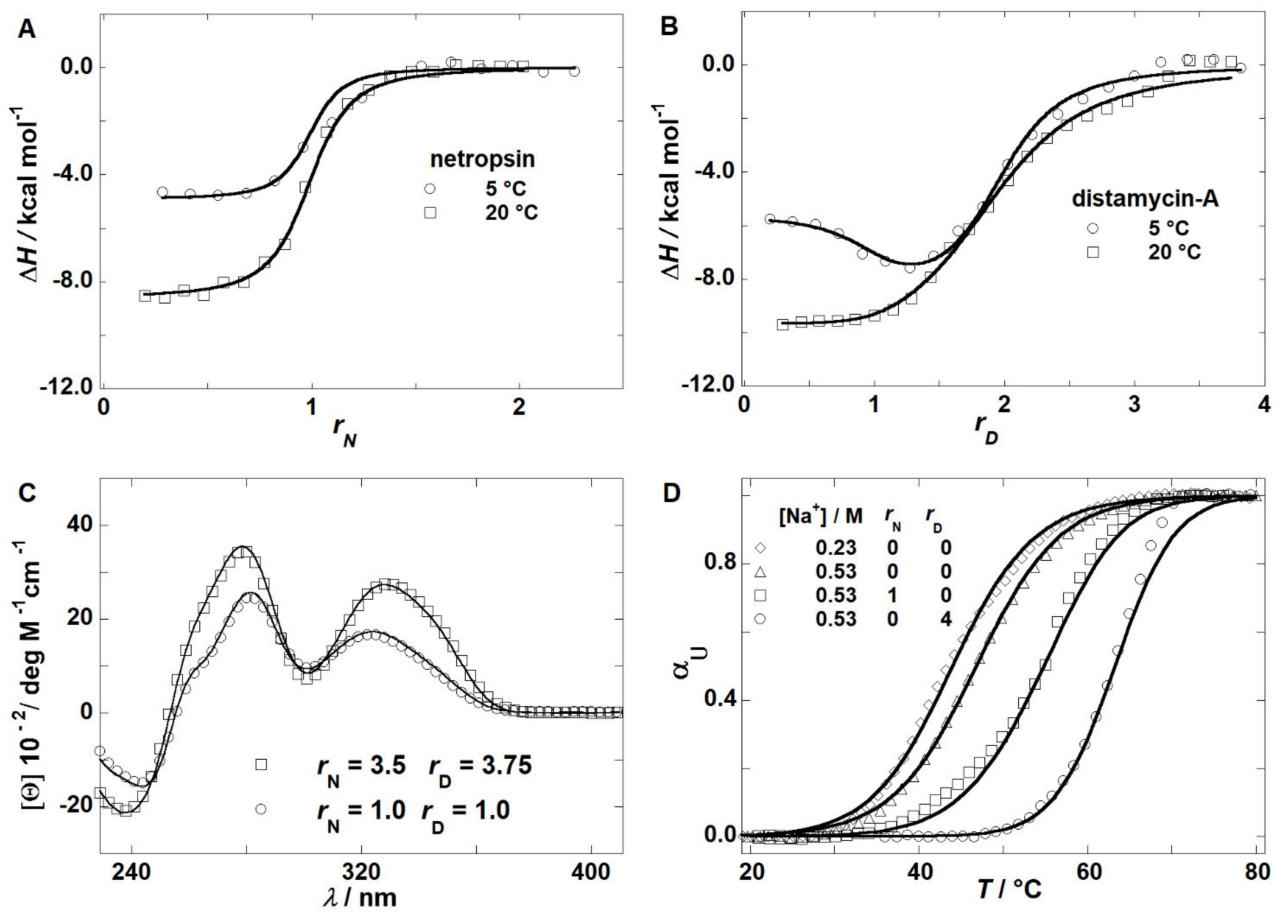
ITC isotherms accompanying netropsin (panel (**A**)) and distamycin (panel (**B**)) binding to the folded oligonucleotide. CD spectra of the oligonucleotide, netropsin (N), and distamycin (D) mixtures measured at 20 °C (panel (**C**)). UV-melting curves measured in the absence and in the presence of ligands (N or D). Symbols represent experimental data. Lines in panels (**A**,**B**,**D**) (absence of ligands) represent the corresponding best-fit model functions (Equations (1), (2), and (14)), while lines in panels (**C**,**D**) (presence of ligands) represent the calculated CD spectra (Equation (12)) and UV-melting curves (Equation (14)) using the best-fit parameters presented in Table 1.

Calorimetric isotherms resulting from titrating the oligonucleotide (F) solution with netropsin (N) solution are shown in Figure 1A. Plateau at *r* < 1 values followed by a significant Δ*H* magnitude drop at *r* ≈ 1 suggest 1:1 binding of N to F. This is confirmed by a good global fit of the corresponding model function (see for example ref. [22] for a detailed description of the global fitting procedure)

(1) $$\Delta H_{\left(T\right)}=\Delta H_{\mathrm{FN}\left(T\right)}{\left(\frac{\partial \alpha_{\mathrm{FN}}}{\partial r_{N}}\right)}_{\left(p,T\right)}$$

to the experimental isotherms measured at 5 °C and 20 °C at which the oligonucleotide is in the folded state. In Equation (1), $\Delta H_{\mathrm{FN}}$ is considered as the standard enthalpy of the complex formation (F + N → FN) at a given temperature, *T*, and $\left(\partial \alpha_{\mathrm{FN}}/\partial r_{N}\right)$ is the corresponding partial derivative in which $\alpha_{\mathrm{FN}}$ is the fraction of the oligonucleotide included in the FN complex, and *r*_N_ is the molar ratio between the total amount of N and total amount of oligonucleotide present in the measured solution. Fitting resulted in a set of standard thermodynamic parameters for the netropsin binding: Gibbs free energy, Δ*G*_FN_, enthalpy, Δ*H*_FN_, entropy, Δ*S*_FN_, and heat capacity, Δ*C_p_*_,FN_ (Table 1).

The second set of calorimetric isotherms resulting from titrating the folded oligonucleotide (F) solution with distamycin (D) solution are presented in Figure 1B. Significant Δ*H* magnitudes at *r* < 2 values followed by a drop at *r* ≈ 2 suggest a 2:1 binding of D to F. Moreover, the signal differences in *r* regions between 0 < *r* < 1 and 1 < *r* < 2 observed for each individual isotherm suggest the sequential binding of two D molecules to F. This is confirmed by a good global fit of the corresponding model function

(2) $$\Delta H_{\left(T\right)}=\Delta H_{\mathrm{FD}\left(T\right)}{\left(\frac{\partial \alpha_{\mathrm{FD}}}{\partial r_{D}}\right)}_{\left(p,T\right)}+\Delta H_{{\mathrm{FD}}_{2}\left(T\right)}{\left(\frac{\partial \alpha_{{\mathrm{FD}}_{2}}}{\partial r_{D}}\right)}_{\left(p,T\right)}$$

to the experimental isotherms measured at 5 °C and 20 °C. In Equation (2) $\Delta H_{\mathrm{FD}}$ and $\Delta H_{{\mathrm{FD}}_{2}}$ are considered as the standard enthalpies of FD (F + D → FD) and FD_2_ (F + 2D → FD_2_) complex formation, respectively, and ${\left(\partial \alpha_{\mathrm{FD}}/\partial r_{D}\right)}_{⬚}$ and $\left(\partial \alpha_{\mathrm{FD}2}/\partial r_{D}\right)$ are the corresponding partial derivatives in which $\alpha_{\mathrm{FD}}$ and $\alpha_{\mathrm{FD}2}$ are the fractions of the oligonucleotide included in the FD and FD_2_ complexes, and *r*_D_ is the molar ratio between total amount of D and total amount of oligonucleotide present in the measured solution. Global fitting resulted in a set of standard thermodynamic parameters of distamycin binding: Gibbs free energies, Δ*G*_FD_ and $\Delta G_{{\mathrm{FD}}_{2}}$, enthalpies, ΔH_FD_ and $\Delta H_{{\mathrm{FD}}_{2}}$, entropies, ΔS_FD_ and $\Delta S_{{\mathrm{FD}}_{2}}$ and heat capacities, Δ*C_p_*_,FD_ and $\Delta C_{p,{\mathrm{FD}}_{2}}$ (Table 1). While the parameters Δ*F*_FD_ (*F* = *G, H, S, C_p_*) reflect the binding of the first D molecule per se (Δ*F*_D1_ = Δ*F*_FD_), the parameters reflecting the binding of the second distamycin molecule (FD + D → FD_2_) were calculated as Δ*F*_D2_ = $\Delta F_{{\mathrm{FD}}_{2}}-\Delta F_{{\mathrm{FD}}_{⬚}}$.

The values of thermodynamic parameters are in agreement with those reported for similar systems [19,23] and show that in the measured temperature range, the binding of netropsin, the first distamycin molecule, and the second one is an exothermic (Δ*H*_j_ < 0, j = FN, D1, D2) enthalpy-driven process (|Δ*H*_j_| > |*T*Δ*S*_j_|). Δ*H*_j_ < 0 can be mainly attributed to the establishment of short-range non-covalent ligand interactions with the AAAAA binding site, while slightly more negative Δ*H*_D2_ in comparison to Δ*H*_D1_ most likely reflects additional van der Waals interactions between the two stacked distamycin molecules. *T*Δ*S*_j_ mainly reflects the favorable contribution due to dehydration and unfavorable contributions due to the reduced translational, rotational, and conformational freedom of F and N molecules. Table 1 shows that at 20 °C, these contributions compensate for each other to a high extent resulting in a relatively small *T*Δ*S*_j_ value except for binding of the second distamycin molecule, for which the unfavorable entropy contributions overcompensate the favorable contribution due to dehydration. The observed negative value of binding heat capacities (Δ*C_p_*_,j_ < 0) additionally emphasizes the importance of dehydration as an important driving force of netropsin and distamycin binding [19]. Δ*C_p_*_,j_ values of around −0.2 kcal mol^−1^ K^−1^ agree well with those estimated on the basis of changes in solvent-accessible surface areas upon binding using 3D structures of netropsin–DNA and distamycin–DNA complexes [24].

### 2.2. Thermodynamics of (un)Folding

Normalized UV-melting curves measured in the absence and presence of netropsin and distamycin are presented in Figure 1D. The curves obtained at different salt concentrations in the absence of ligands were analyzed in terms of the reversible two-state model of (un)folding (F ↔ U). A good global fit of the calculated fraction of the unfolded oligonucleotide, $\alpha_{U}$ (Equation (14), see ref. [24] for details), to the experimental curves enabled us to obtain the following standard thermodynamic parameters of oligonucleotide folding: Gibbs free energy, Δ*G*_F_, enthalpy, Δ*H*_F_, entropy, Δ*S*_F_, and heat capacity, Δ*C_p_*_,F_ (Table 1). As expected, folding at 20 °C is an enthalpy-driven process (Δ*H*_F_ < 0) accompanied by unfavorable entropy contribution *T*Δ*S*_F_ < 0 and negative heat capacity changes. The obtained Δ*G*_F_, Δ*H*_F_, and Δ*S*_F_ values are in general agreement with the corresponding ones estimated via the nearest-neighbor approach [25] or by Privalov group [26], and the presented Δ*C_p_*_,F_ value agrees well the reported experimental and structure-based estimates [26,27]. The estimated no. of Na^+^ ions bound to the oligonucleotide upon its folding (Table 1) corresponds well to the those determined experimentally for some similar hairpin-forming sequences [28].

### 2.3. Thermodynamic Parameters of Binding and Folding Define the Species Populations

Each transition step j (j = F, FN, FD, FD_2_; see Scheme 1) can be described in terms of the corresponding changes of three standard thermodynamic parameters that are independent of added salt concentration. The standard Gibbs free energy and standard enthalpy depend on temperature and are presented at the reference temperature *T*_0_ = 293.15 K = 20 °C as $\Delta G_{\left(T_{0}\right)}$ and $\Delta H_{j\left(T_{0}\right)}$. The standard heat capacity, $\Delta C_{P,j}$, is considered to be temperature independent in the studied temperature range. These three parameters define the standard enthalpy, $\Delta H_{j\left(T\right)}$, standard Gibbs free energy of transition,$\;\Delta G_{j\left(T\right)}$, and standard entropy of transition, $\Delta S_{j\left(T\right)}$, at any *T* through the Kirchhoff’s law

(3) $$[\partial \left(\Delta H_{j}/T\right)/\partial T{]}_{p}=\Delta C_{P,j}\Rightarrow H_{j\left(T\right)}=\Delta H_{j\left(T_{0}\right)}+\Delta C_{P,j}[T-T_{0}]$$

the Gibbs-Helmholtz equation

(4) $$[\partial \left(\Delta G_{j}/T\right)/\partial T{]}_{p}=-\Delta H_{j}/T^{2}\Rightarrow \;\Delta G_{j\left(T\right)}=\Delta G_{j\left(T_{0}\right)}\left[T/T_{0}\right]+\Delta H_{j\left(T_{0}\right)}\left[1-T/T_{0}\right]+\Delta C_{P,j}[T-T_{0}-Tln\left(T/T_{0}\right)$$

and the general relation

(5) $$\Delta G_{j\left(T\right)}=\Delta H_{j\left(T\right)}-T\Delta S_{j\left(T\right)}.$$

DNA oligonucleotide folding and each ligand binding event can be described in terms of the apparent standard Gibbs free energy, $\Delta G_{\left(T,\mathrm{Na}\right)}$, which depends on *T* and cation (Na^+^) concentration. Its relation to the standard thermodynamic $\Delta G_{\left(T\right)}$ is given by^10^

(6) $$\Delta G_{j\left(T,\mathrm{Na}\right)}=\Delta G_{j\left(T\right)}-n_{\mathrm{Na},j}RT\ln[{\mathrm{Na}}^{+}]$$

where *n*_Na,j_ represents the apparent number of Na^+^ ions bound/released in transition step j and is considered to be temperature independent. [Na^+^] represents equilibrium molar concentrations of unbound Na^+^ ions normalized to 1 M concentration in the reference (standard) state. According to Equation (6), four parameters ($\Delta G_{j\left(T_{0}\right)}$, $\Delta H_{j\left(T_{0}\right)}$, $\Delta C_{P,j}$, *n*_Na,j_) are needed for calculating $\Delta G_{j\left(T,\mathrm{Na}\right)}$ and thereby for the thermodynamic description of each transition step at different *T* and [Na^+^]. Namely, $\Delta G_{j\left(T,\mathrm{Na}\right)}$ defines the corresponding apparent equilibrium constant

(7) $$K_{j\left(T,\mathrm{Na}\right)}=\exp(-\Delta G_{j\left(T,\mathrm{Na}\right)}/RT)$$

which is for each transition step j related to equilibrium normalized species concentrations, $\alpha_{i}$, as:

(8) $$K_{F}=\frac{\alpha_{F}}{\alpha_{U}},\;K_{\mathrm{FN}}=\frac{\alpha_{\mathrm{FN}}}{\alpha_{F}\alpha_{N}C},\;K_{\mathrm{FD}}=\frac{\alpha_{\mathrm{FD}}}{\alpha_{F}\alpha_{D}C}=K_{\mathrm{FD}1},\;K_{{\mathrm{FD}}_{2}}=\frac{\alpha_{{\mathrm{FD}}_{2}}}{\alpha_{F}\alpha_{D}^{2}C^{2}}=K_{\mathrm{FD}1}K_{\mathrm{FD}2}.$$

The normalized concentrations are defined as *α*_i_ = [i]/*C*. where [i] represents equilibrium molar concentrations of i (i = U, F, N, D, FN, FD, FD_2_), while *C* represents the total molar concentration of DNA. For i = U, F, FN, FD, FD_2_, the normalized species concentrations *α*_i_ represent the fraction of total DNA included in species i. If the parameters ($\Delta G_{j\left(T_{0}\right)}$, $\Delta H_{j\left(T_{0}\right)}$, $\Delta C_{P,j}$, *n*_Na,j_), and thereby $K_{j\left(T,\mathrm{Na}\right)}$, are known for each transition step and since

(9) $$\alpha_{U}+\alpha_{F}+\alpha_{\mathrm{FN}}+\alpha_{\mathrm{FD}}+\alpha_{{\mathrm{FD}}_{2}}=\alpha_{U}K_{F}\left(1+K_{\mathrm{FN}}\alpha_{N}C+K_{\mathrm{FD}}\alpha_{D}C+K_{{\mathrm{FD}}_{2}}\alpha_{D}^{2}C^{2}\right)=1$$

(10) $$\alpha_{N}+\alpha_{\mathrm{FN}}=\alpha_{N}(1+K_{F}K_{\mathrm{FN}}\alpha_{U}C)=r_{N}$$

(11) $$\alpha_{D}+\alpha_{\mathrm{FD}}+2\alpha_{{\mathrm{FD}}_{2}}=\alpha_{D}\left(1+K_{F}K_{\mathrm{FD}}\alpha_{U}C+2K_{F}K_{{\mathrm{FD}}_{2}}\alpha_{U}\alpha_{D}^{⬚}C^{2}\right)=r_{D},$$

one may calculate the fractions $\alpha_{F},\;\alpha_{\mathrm{FN}},\alpha_{\mathrm{FD}},\;\mathrm{and}\;\alpha_{\mathrm{FD}2}$ from Equation (8) by solving the system of Equations (9)–(11) for $\alpha_{U}$, $\alpha_{N}$, and $\alpha_{D}$ at any *r*_N_, *r*_D_, *T*, and [Na^+^].

### 2.4. Model Predicted Species Populations Are in Accordance with Experimental Observations

Specifically, for netropsin (N) binding at T ≤ 20 °C and fixed [Na^+^] in the absence of distamycin ($\alpha_{D}$ = 0), the parameters $\Delta G_{\mathrm{FN}\left(T_{0}\right)}$, $\Delta H_{\mathrm{FN}\left(T_{0}\right)}$, and $\Delta C_{P,\mathrm{FN}}$ (Table 1) define $\Delta H_{\mathrm{FN}}$ at any *T* through Equation (3), and *α*_F_, *α*_N_, and *α*_FN_ at any *r*_N_, and *T* through Equations (4)–(10) and consequently, the corresponding model function for the description of ITC data (Equation (1)). Similarly, for distamycin (D) binding in the absence of netropsin ($\alpha_{N}$ = 0), the parameters $\Delta G_{\mathrm{FD}\left(T_{0}\right)}$, $\Delta H_{\mathrm{FD}\left(T_{0}\right)}$, $\Delta C_{P,\mathrm{FD}}$, $\Delta G_{{\mathrm{FD}}_{2}\left(T_{0}\right)}$, $\Delta H_{{\mathrm{FD}}_{2}\left(T_{0}\right)}$, and $\Delta C_{P,{\mathrm{FD}}_{2}}$ (Table 1) define $\Delta H_{\mathrm{FD}}$ and $\Delta H_{{\mathrm{FD}}_{2}}$ at any T through Equation (3), and α_F_, α_D_, α_FD_, and $\alpha_{{\mathrm{FD}}_{2}}$ at any *r*_D_ and *T* through Equations (4)–(9) and (11), and consequently the corresponding model function for the description of ITC data (Equation (2)). For netropsin and distamycin binding, the model functions show good agreement with experimental data (Figure 1A,B), indicating that the best-fit parameter values are appropriate for describing the binding of the two ligands to the folded oligonucleotide.

**Figure 2:**
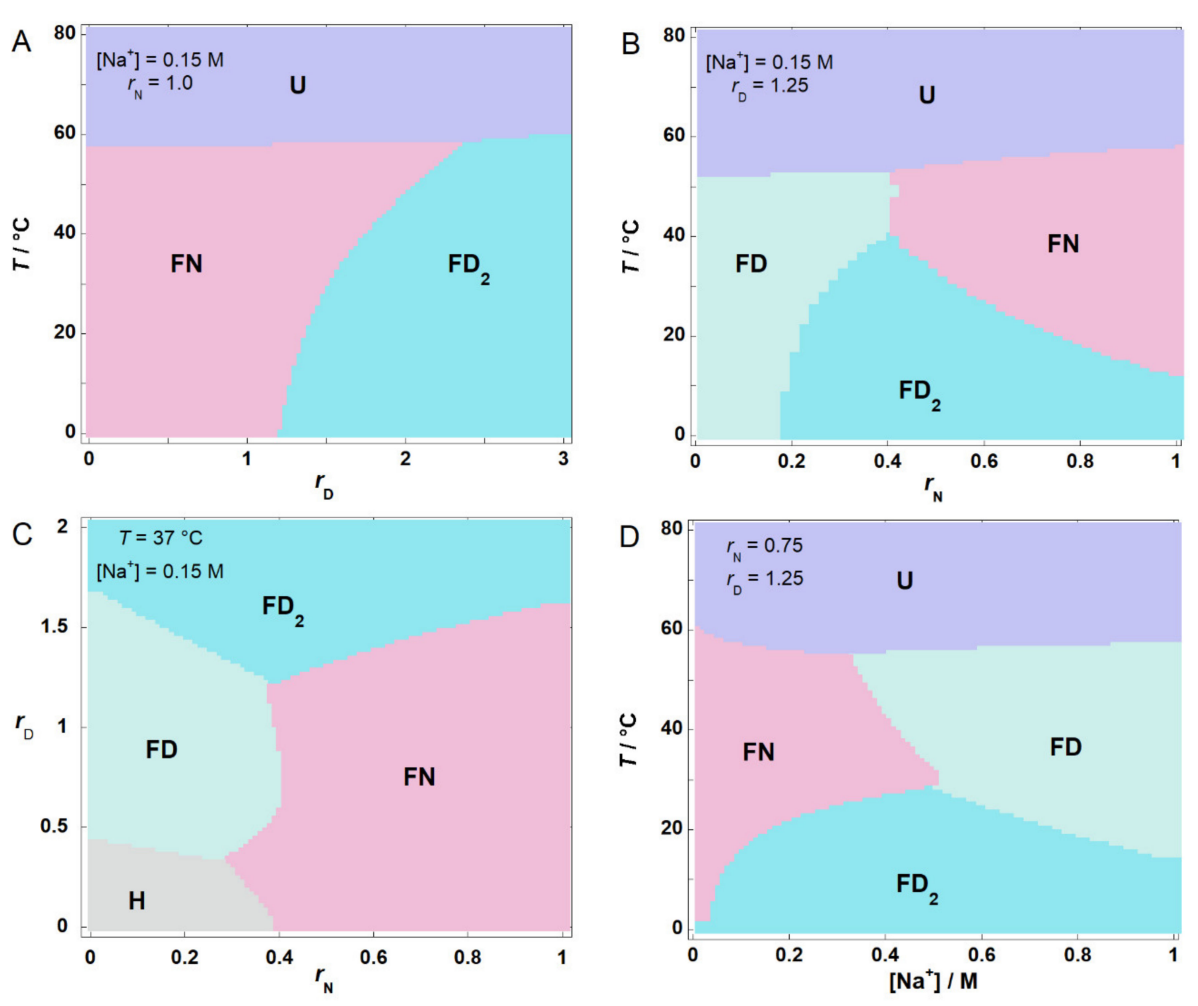
Phase diagrams showing the most populated model predicted DNA species (Scheme 1) at *C*_DNA_ = 5 μM. *T* versus given *r*_D_ diagram (panel **A**), *T* versus *r*_N_ diagram (panel **B**), *r*_D_ versus *r*_N_ diagram (panel **C**), and *T* versus [Na^+^] diagram (panel **D**).

To check if the parameters describe correctly the behavior of the folded DNA (F) in the simultaneous presence of netropsin (N) and distamycin (D), we performed CD-spectroscopy measurements in F, N, and D mixtures (Figure 2C). The measured CD signal can be at any wavelength, and *T* is presented as a linear combination of characteristic signals (molar ellipticities) of species i, ${\left[\theta \right]}_{i}$, weighted by the corresponding fractions *α*_i_

(12) $$\left[\theta \right]=\sum_{i}{\left[\theta \right]}_{i}\alpha_{i}={\left[\theta \right]}_{F}\alpha_{F}+{\left[\theta \right]}_{\mathrm{FN}}\alpha_{\mathrm{FN}}+{\left[\theta \right]}_{\mathrm{FD}}\alpha_{\mathrm{FD}}+{\left[\theta \right]}_{{\mathrm{FD}}_{2}}\alpha_{{\mathrm{FD}}_{2}}$$

Note that the unbound ligands are not optically active (${\left[\theta \right]}_{N}={\left[\theta \right]}_{D}=0$) and that at the measured conditions, the fraction of the unfolded form is negligible ($\alpha_{U}\approx$ 0). Molar ellipticity ${\left[\theta \right]}_{F}$ was obtained from measuring the CD spectra of DNA in the absence of ligands ($\alpha_{\mathrm{FN}}=\alpha_{\mathrm{FD}}=\alpha_{{\mathrm{FD}}_{2}}=0$). Next, we analyzed the CD spectra measured in the presence of N and absence of D ($\alpha_{\mathrm{FD}}=\alpha_{{\mathrm{FD}}_{2}}=0$). The fractions $\alpha_{F}$ and $\alpha_{\mathrm{FN}}$ were calculated for given *r*_N_ using parameters obtained from ITC model analysis (Table 1), which enables estimation of ${\left[\theta \right]}_{\mathrm{FN}}$ from Equation (12). Similarly, we analyzed CD spectra measured in the presence of D and absence of N ($\alpha_{\mathrm{FN}}=0$). The fractions $\alpha_{F}$, $\alpha_{\mathrm{FD}}$, and $\alpha_{{\mathrm{FD}}_{2}}$ were calculated for given *r*_D_ using parameters obtained from ITC model analysis (Table 1), which enables estimation of ${\left[\theta \right]}_{\mathrm{FD}}$ and ${\left[\theta \right]}_{{\mathrm{FD}}_{2}}$ from Equation (12). Finally, we analyzed CD spectra of F, N, and D mixtures. The fractions *α*_i_ were calculated for different *r*_N_ and *r*_D_ by solving the system of Equations (9)–(11) using a given set of thermodynamic parameters (Table 1). The estimation of ${\left[\theta \right]}_{i}$ and α_i_ presented above enabled us to calculate $\left[\theta \right]$ from Equation (12). The calculated $\left[\theta \right]$ shows excellent agreement with the measured CD spectra of the mixtures, confirming that the obtained parameters are appropriate to describe the behavior of the folded DNA (F) in the simultaneous presence of netropsin (N) and distamycin (D).

To check whether the combination of parameters describing oligonucleotide (un)folding and ligand binding (Table 1) successfully describes the observed ligand-induced thermal stabilization of DNA and thus its behavior in the presence of ligands at various temperatures, we performed UV-melting experiments. Similarly to the CD signal, the measured UV light absorption, A, can be at a given wavelength and any *T* analyzed in terms of a linear combination

(13) $$A=\sum_{i}A_{i}\alpha_{i}=A_{F}\alpha_{F}+A_{\mathrm{FN}}\alpha_{\mathrm{FN}}+A_{\mathrm{FD}}\alpha_{\mathrm{FD}}+A_{{\mathrm{FD}}_{2}}\alpha_{{\mathrm{FD}}_{2}}+A_{U}\alpha_{U}$$

in which $A_{i}$ represents the characteristic absorption of species i at fixed total DNA concentration *C*. In the absence of ligands ($\alpha_{\mathrm{FN}}=\alpha_{\mathrm{FD}}=\alpha_{{\mathrm{FD}}_{2}}=0$), the measured absorption can be normalized as

(14) $$\frac{A-A_{\mathrm{fold}}}{A_{U}-A_{\mathrm{fold}}}=\alpha_{U}.$$

In Equation (14), A_fold_ ($=A_{F}$) is obtained as a pre-transition low-temperature baseline, and $A_{U}$ is obtained as a post-transition high-temperature baseline. Experimental $\alpha_{U}$ estimated from Equation (14) is presented in Figure 1D. As mentioned above, fitting of the $\alpha_{U}$ calculated for different *T* and [Na^+^] from Equations (3)–(9) resulted in the thermodynamic parameters of oligonucleotide folding presented in Table 1. Next, we use Equation (14) to analyze the observed monophasic melting curves accompanying the unfolding of netropsin or distamycin-bound DNA. Since we monitored DNA that is sufficiently saturated with the bound ligand, A_fold_ in Equation (14) can be approximated as a pre-transition baseline representing $A_{\mathrm{FN}}\;$(netropsin) or $A_{{\mathrm{FD}}_{2}}$ (distamycin). In addition, $\alpha_{U}$, as a function of *T* was calculated for *r*_D_ = 4 or *r*_N_ = 1 using the parameter set given in Table 1 and Equations (3)–(11). Good agreement between the calculated and experimentally estimated $\alpha_{U}$ (Figure 1D) strongly suggests that DNA oligonucleotide behavior at various temperatures in the absence and in the presence of ligands can be successfully described using the parameter values presented in Table 1.

### 2.5. Phase Diagrams Illustrate the Nature of Conditional Cooperativity

The obtained set of parameters (Table 1) was used for calculation of the DNA species fractions in the solution as a function of *r*_N_, *r*_D_, *T*, and [Na^+^] through Equations (3)–(11). The most populated species at given *r*_N_, *r*_D_, *T*, and [Na^+^] is considered as a pseudophase. The corresponding 2D (*T* versus *r*_D_, *T* versus *r*_N_, *r*_D_ versus *r*_N_, *T* versus [Na^+^]) phase diagrams were constructed by assigning areas in the phase space to the most populated species in that area, i.e., pseudophase (Figure 2). Phase curves (borders) and triple points represent states in which two (curves) or three (triple points) of the most populated species (pseudo phases) are equally populated. These diagrams indicate the possible macroscopic pathways of the model DNA binding and (un)folding.

Perhaps the most intuitive behavior of the interacting system is presented in the *T* versus *r*_D_ diagram (Figure 2A) that shows that at *r*_N_ = 1, [Na^+^] = 0.15 M, and *T* < 60 °C, an increasing of distamycin concentration induces the formation of the FD_2_ complex. This happens because two distamycin molecules are able to displace one bound netropsin molecule from the binding site. With increasing *T*, more distamycin is needed for the displacement, since $\Delta H_{j}<0$ and $\left|\Delta H_{{\mathrm{FD}}_{2}}\right|$ > $\left|\Delta H_{{\mathrm{FN}}_{⬚}}\right|$, and thus, $K_{{\mathrm{FD}}_{2}}$ decreases with T more rapidly than $K_{\mathrm{FN}}$.

The nature of conditional cooperativity is presented in the *T* versus *r*_N_ diagram (Figure 2B) that shows that at *r*_D_ = 1.25, [Na^+^] = 0.15 M, and *T* < 40 °C, an increasing of netropsin concentration induces the formation of the FD_2_ complex (i.e., cooperativity). This happens because N displaces D from FD complexes, and the displaced D is able to bind to the remaining FD complexes forming FD_2_, which at *r*_N_ > 0.2 becomes the most populated species, while FN dominates at higher *r*_N_ > 0.5—high netropsin concentrations reduce distamycin binding cooperativity. The FD_2_ region is larger at lower temperatures, since $\Delta H_{j}<0$ and $|\Delta H_{{\mathrm{FD}}_{2}}|\;$> $\left|\Delta H_{FN}\right|$≈ $\left|\Delta H_{{\mathrm{FD}}_{⬚}}\right|$, meaning that $K_{{\mathrm{FD}}_{2}}$ decreases with T more significantly than $K_{\mathrm{FN}}$ or $K_{\mathrm{FD}}$.

Similarly to the *T* versus *r*_N_ diagram, the *r*_D_ versus *r*_N_ diagram (Figure 2C) shows that at 37 °C, [Na^+^] = 0.15 M, and *r*_D_ ≈1.5, an increasing of netropsin concentration induces the distamycin cooperative binding. At 0.2 < *r*_N_ < 0.6, FD_2_ is the most populated species, while FN becomes the predominant species at higher *r*_N_ > 0.6. Interestingly, increasing distamycin concentration at *r*_N_ ≈ 0.4 first induces the formation of FD but 1 < *r*_D_ < 1.5 FN again becomes the most populated species due to distamycin binding cooperativity (i.e., the formation of FD_2_).

The *T* versus [Na^+^] diagram (Figure 2D) shows for example that at *r*_N_ = 0.75 and *r*_D_ = 1.25, increasing of [Na^+^] at *T* ≈ 20 °C first induces distamycin cooperative binding, while further increasing of [Na^+^] leads to its reduction (FD becomes the most populated species). This is because different amounts of Na^+^ ions are released from F upon N or D binding ($n_{\mathrm{Na},\mathrm{FN}}$ > $n_{{\mathrm{Na},\mathrm{FD}}_{2}}$ > $n_{\mathrm{Na},\mathrm{FD}}$). Since Na^+^ ions are released upon ligand binding, increasing [Na^+^] destabilizes FN, FD, and FD_2_ complexes (Na^+^ ions compete for binding with ligands). However, since $n_{\mathrm{Na},\mathrm{FN}}$ > $n_{{\mathrm{Na},\mathrm{FD}}_{2}}$ > $n_{\mathrm{Na},\mathrm{FD}}$, FN is destabilized more than FD_2_ and FD_2_ is destabilized more than FD, leading to the observed FN → FD_2_ → FD pathway.

## 3. Conclusions

In summary, we demonstrate how phase diagrams can be obtained from the calorimetric and spectroscopic data analysis and used for visualization of the pathways of DNA ligand binding and conformational transitions. Phase diagrams clearly show the nature of the observed distamycin binding cooperativity that can be induced or reduced by netropsin or/and Na^+^ ions due to the linkage of the multiple binding equilibria. Due to differences in binding enthalpies, the cooperative binding is more pronounced at lower temperatures. Our study emphasizes that the conditional cooperativity is a phenomenon that is not exclusive only for biological macromolecules but can also be observed in interacting systems involving small molecules.

## 4. Materials and Methods

### 4.1. DNA and Ligand Preparation

Purified (HPLC) samples of the oligonucleotide 5′-GAAAAAACCCCCCTTTTTC-3′ (Scheme 1) were obtained from Invitrogen, while netropsin (N) and distamycin-A (D) were purchased from Sigma Aldrich. The oligonucleotide concentration in the measured solution was determined using UV absorption spectroscopy using a nearest neighbor estimation of the extinction coefficient [29] for single-strand DNA (*ε*_U,260_ = 138,500 M^−1^ cm^−1^) and the absorbance at 260 nm of thermally denatured oligonucleotide extrapolated back to 20 °C. Concentrations of netropsin (N) and distamycin (D) were determined from the measured absorbance at 296 nm (N) and 303 nm (D) at 25 °C using the published extinction coefficient (*ε*_N,296_ = 21,500 M^−1^ cm^−1^, *ε*_N,296_ = 34,000 M^−1^ cm^−1^) [19]. Oligonucleotide and ligand solutions were prepared in phosphate buffer solution (10 mM Na-phosphate, 200 mM (CD, UV) or 500 mM (ITC) NaCl, 1 mM EDTA, pH = 7.0). Prior to each calorimetric and spectroscopic measurement, oligonucleotide and ligand solutions were degassed for about 20 min.

### 4.2. Isothermal Titration Calorimetry (ITC)

ITC experiments were performed at 5 °C and 20 °C by titrating a ligand solution (*c*_ligand_ ≈ 50 μM) into an oligonucleotide solution (*c*_DNA_ ~3.5 μM, V = 1.386 mL) at using a VP-ITC isothermal titration calorimeter from Microcal Inc. (Northampton, MA, USA). The area under the peak following each injection of L solution was obtained by integration of the raw signal, corrected for the corresponding heat of dilution, and expressed per mole of added L per injection, to give the enthalpy of interaction, $\Delta H_{\left(T\right)}$. The obtained experimental isotherms (Figure 1A,B) data were modeled as described above.

### 4.3. UV-Absorption Spectroscopy (UV)

All absorbance measurements were performed in a Cary Bio 100 UV spectrophotometer (Varian, Australia) equipped with a thermoelectrically controlled cell holder. Consecutive UV melting scans (heating rate of 1 °C min^−1^) measured at 260 nm and different oligonucleotide concentrations measured show only one major unfolding transition (*T*_m_ ≈ 44 °C at 200 mM NaCl) that is independent of oligonucleotide concentration, indicating that at low temperatures, the oligonucleotide appears in the folded hairpin (F) form (Scheme 1). To obtain thermodynamic parameters of (un)folding, UV melting experiments were performed in a 1 cm cuvette (200 mM NaCl or 500 mM NaCl, *c*_DNA_ ≈3.5 μM) and analyzed in terms of the reversible two-state model of (un)folding as described above [24].

### 4.4. Circular Dichroism Spectroscopy (CD)

CD spectra were measured with an AVIV Model 62A DS spectropolarimeter (Aviv Associates, Lakewood, NJ, USA) equipped with a thermoelectrically controlled cell holder. CD spectra were recorded between 220 and 420 nm in a 1 cm cuvette (*c*_DNA_ ≈10 μM, 200 mM NaCl, 20 °C). Molar ellipticity, $\left[\theta \right]$, was obtained by normalizing the measured spectra with subtracted baseline (buffer contribution) to 1 M DNA concentration and 1 cm optical pathlength.

## Figures and Tables

**Table 1:** Thermodynamic parameters characterizing the observed folding and binding steps (Scheme 1) at 20 °C and 500 mM concentration of Na^+^ ions.

t	U → F	F + N → FN	F + D → FD	FD + D →FD_2_	F + 2D → FD_2_
$\Delta G_{j}$/ kcal mol^−1^	−3.1 ± 0.2	−9.9 ± 0.1	−10.2 ± 0.1	−8.3 ± 0.1	−18.5 ± 0.2
$\Delta H_{j}$/ kcal mol^−1^	−34.1 ± 0.2	−8.6 ± 0.3	−9.7 ± 0.4	−10.9 ± 0.6	−20.6 ± 1.0
$T\Delta S_{j}$/ kcal mol^−1^	−31.0 ± 0.2	1.3 ± 0.4	0.5 ± 0.5	−2.6 ± 0.7	−2.1 ± 1.2
$\Delta C_{p,j}$/ kcal mol^−1^K^−1^	−0.26 ± 0.05	−0.25 ± 0.02	−0.26 ± 0.04	−0.13 ± 0.5	−0.39 ± 0.09
*n*_Na,j_	−1.18 ± 0.2	1.95 ± 0.5 ^a^	0.62 ± 0.25 ^a^	0.63 ± 0.25 ^a^	1.25 ± 0.5 ^a^

^a^ Calculated based on ref. [23].

## Data Availability

Data are available on request from the corresponding author.

## References

[1] Overgaard M., Borch J., Jørgensen M.G., Gerdes K. (2008). Messenger RNA interferase RelE controls relBE transcription by condi-tional cooperativity. Mol. Microbiol..

[2] Garcia-Pino A., Balasubramanian S., Wyns L., Gazit E., De Greve H., Magnuson R.D., Charlier D., van Nuland N.A., Loris R. (2010). Allostery and Intrinsic Disorder Mediate Transcription Regulation by Conditional Cooperativity. Cell.

[3] Garcia-Pino A., De Gieter S.S., Talavera A.A., De Greve H., Efremov R., Loris R. (2016). An intrinsically disordered entropic switch determines allostery in Phd–Doc regulation. Nat. Chem. Biol..

[4] Vandervelde A., Drobnak I., Hadži S., Sterckx Y., Welte T., De Greve H., Charlier D., Efremov R., Loris R., Lah J. (2017). Molecular mechanism governing ratio-dependent transcription regulation in the ccdAB operon. Nucleic Acids Res..

[5] De Bruyn P., Girardin Y., Loris R. (2021). Prokaryote toxin–antitoxin modules: Complex regulation of an unclear function. Protein Sci..

[6] Chaires J.B. (1998). Drug-DNA interactions. Curr. Opin. Struct. Biol..

[7] Smidlehner T., Badovinac M., Piantanida I. (2018). Pyrene-cyanine conjugates as multipurpose fluorescent probes for non-covalent recognition of ds-DNA, RNA and proteins. New J. Chem..

[8] Ban Ž., Žinić B., Vianello R., Schmuck C., Piantanida I. (2017). Nucleobase–Guanidiniocarbonyl-Pyrrole Conjugates as Novel Fluo-rimetric Sensors for Single Stranded RNA. Molecules.

[9] Radić-Stojković M., Piotrowski P., Schmuck C., Piantanida I. (2015). Short, rigid linker between pyrene and guanidiniocarbon-yl-pyrrole induced new set of spectroscopic responses to ds-DNA secondary structure. Org. Biomol. Chem..

[10] White S.K., Szewczyk J.W., Turner J.M., Baird E.E., Dervan P.B. (1998). Recognition of the four Watson–Crick base pairs in the DNA minor groove by synthetic ligands. Nature.

[11] Geierstanger B.H., Wemmer D.E. (1995). Complexes of the minor groove of DNA. Annu. Rev. Biophys. Biomol. Struct..

[12] Neidle S. (2002). Nucleic Acid Structure and Recognition.

[13] Kopka M.L., Yoon C., Goodsell D., Pjura P., Dickerson R.E. (1985). The molecular origin of DNA-drug specificity in netropsin and distamycin. Proc. Natl. Acad. Sci. USA.

[14] Marky L.A., Breslauer K.J. (1987). Origins of netropsin binding affinity and specificity: Correlations of thermodynamic and structural data. Proc. Natl. Acad. Sci. USA.

[15] Rentzeperis D., Marky L.A. (1993). Netropsin binding as a thermodynamic probe of the grooves of parallel DNA. J. Am. Chem. Soc..

[16] Pelton J.G., Wemmer D.E. (1990). Binding modes of distamycin A with d(CGCAAATTTGCG)2 determined by two-dimensional NMR. J. Am. Chem. Soc..

[17] Capobianco M.L., Colonna F.P., Forni A., Gabresi A., Lotti S., Moretti I., Samori B., Tondelli L. (1991). Interactions of nucleic acids with distamycins. Binding of Dst-3 to d(CGTTTAAACG)2 and d(CGTACGTACG). Nucleic Acids Res..

[18] Pelton J.G., Wemmer D.E. (1989). Structuralcharacterization of a 2:1 distamycin Ad(CGCAAATTGGC) complex by two-dimensional NMR. Proc. Natl. Acad. Sci. USA.

[19] Rentzeperis D., Marky L.A., Dwyer T.J., Geierstanger B.H., Pelton J.G., Wemmer D.E. (1995). Interaction of minor groove ligands to an AAATT/AATTT site: Correlation of thermodynamic characterization and solution structure. Biochemistry.

[20] Chen F.-M., Sha F. (1998). Circular Dichroic and Kinetic Differentiation of DNA Binding Modes of Distamycin. Biochemistry.

[21] Lah J., Vesnaver G. (2000). Binding of Distamycin A and Netropsin to the 12mer DNA Duplexes Containing Mixed AT·GC Sequences with At Most Five or Three Successive AT Base Pairs. Biochemistry.

[22] Lah J., Drobnak I., Dolinar M., Vesnaver G. (2008). What drives the binding of minor groove-directed ligands to DNA hairpins?. Nucleic Acids Res..

[23] Lah J., Vesnaver G. (2004). Energetic Diversity of DNA Minor-groove Recognition by Small Molecules Displayed Through Some Model Ligand-DNA Systems. J. Mol. Biol..

[24] Drobnak I., Vesnaver G., Lah J. (2010). Model-Based Thermodynamic Analysis of Reversible Unfolding Processes. J. Phys. Chem. B.

[25] Santa Lucia J., Hicks D. (2004). The thermodynamics of DNA structural motifs. Annu. Rev. Biophys. Biomol. Struct..

[26] Privalov P.L., Crane-Robinson C. (2018). Forces maintaining the DNA double helix and its complexes with transcription factors. Prog. Biophys. Mol. Biol..

[27] Hadži S., Lah J. (2021). Origin of heat capacity increment in DNA folding: The hydration effect. BBA Gen. Subj..

[28] Lah J., Seručnik M., Vesnaver G. (2011). Influence of a Hairpin Loop on the Thermodynamic Stability of a DNA Oligomer. J. Nucleic Acids.

[29] Cantor C.R., Warshaw M.M., Shapiro H. (1970). Oligonucleotide interactions. Circular dichroism studies of the conformation of deoxyoligonucleotides. Biopolymers.

